# Protein–DNA/RNA Interactions: An Overview of Investigation Methods in the -Omics Era

**DOI:** 10.1021/acs.jproteome.1c00074

**Published:** 2021-05-07

**Authors:** Flora Cozzolino, Ilaria Iacobucci, Vittoria Monaco, Maria Monti

**Affiliations:** † Department of Chemical Sciences, University Federico II of Naples, Strada Comunale Cinthia, 26, 80126 Naples, Italy; ‡ CEINGE Advanced Biotechnologies, Via G. Salvatore 486, 80145 Naples, Italy; § Interuniversity Consortium National Institute of Biostructures and Biosystems (INBB), Viale Medaglie d’Oro, 305-00136 Rome, Italy

**Keywords:** proteomics, mass spectrometry, DNA−protein interactions, RNA−protein interactions, pull-down, EMSA, ChIP, CRISPR-Cas9

## Abstract

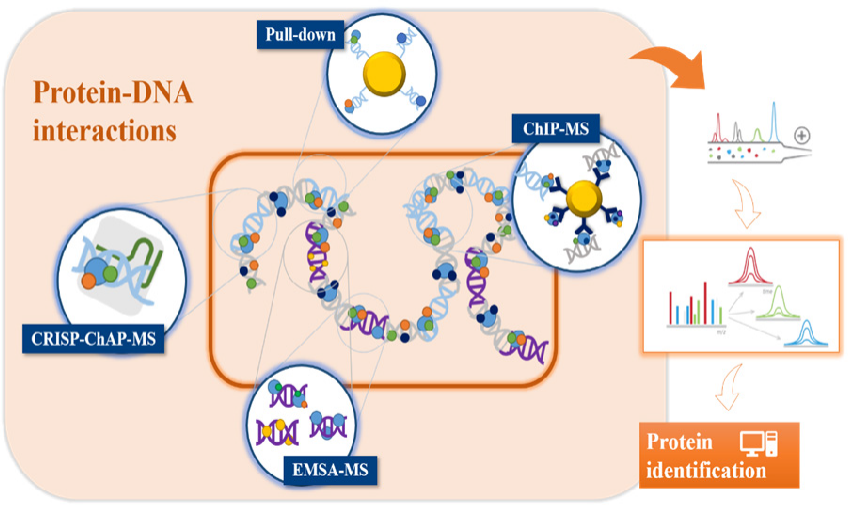

The fields of application of functional proteomics are not limited to the study of protein–protein interactions; they also extend to those involving protein complexes that bind DNA or RNA. These interactions affect fundamental processes such as replication, transcription, and repair in the case of DNA, as well as transport, translation, splicing, and silencing in the case of RNA. Analytical or preparative experimental approaches, both *in vivo* and *in vitro*, have been developed to isolate and identify DNA/RNA binding proteins by exploiting the advantage of the affinity shown by these proteins toward a specific oligonucleotide sequence. The present review proposes an overview of the approaches most commonly employed in proteomics applications for the identification of nucleic acid-binding proteins, such as affinity purification (AP) protocols, EMSA, chromatin purification methods, and CRISPR-based chromatin affinity purification, which are generally associated with mass spectrometry methodologies for the unbiased protein identification.

## 1 Introduction

“Life is a relationship between molecules, not a property of any one molecule. So is, therefore, disease, which endangers life,” wrote Zuckerkandl and Pauling^1,2^ in their chapter on “Molecular disease, evolution and genic heterogeneity”. Several years have passed, and the molecular mechanisms underlying many diseases and the interactions between molecules in healthy and diseased organisms are still poorly understood and unclear. Therefore, we are still very far from understanding and elucidating the complex network of interactions taking place in living organisms.^3^ In the cell, biological functions are not exerted by single individual proteins, but by transient complexes they form, interacting each other or with other molecules such as nucleic acids^4−7^ and metabolites.^8,9^ Thus, the study of proteins and their interactions is essential to understand their roles within the cell and to elucidate the organization of functional networks.

A complete description of cellular processes is then strictly dependent on a clear definition of the complexes that take part in the molecular mechanisms and the individual protein components involved in these functional entities. The association of an unknown protein with partners belonging to a specific complex involved in a particular mechanism might strongly suggest its biological function.^10^ Furthermore, a detailed description of cell signaling pathways could greatly benefit from the elucidation of interactions *in vivo*.^11^

Modern functional proteomic studies are not solely addressed to the study of protein–protein interactions but also to the investigation of the interactions between multiprotein complexes and nucleic acids, thus to define both the biological functions of specific proteins and their influence on nucleic acids dependent events.^12^ It is well-known that cell processes involving DNA (i.e., replication, transcription, processing, repair, specific package, DNA rearrangement, etc.) as well as RNAs (splicing, transport, translation, silencing, etc.) are the result of the constant interaction between functional nucleic acids and specific proteins.^13^

The evolution selected two different ways of protein–DNA binding: a nonspecific manner, as occurring in histones–DNA interaction, and a very selective and specific mode, in which the protein recognition site is strictly nucleotide sequence-dependent.^14^ The same modalities belong also to RNA–protein interactions, as amply illustrated by Guenther et al. in their work, where the meaning of “specific” and “non-specific” of RNA–protein interactions and their complexity is well illustrated. The authors highlight the presence of specific regions of RNA (RNA Binding Domain) that exclusively bind classes of “specific” proteins as well as proteins that perform as many important functions although in absence of specific binding capabilities.^15,16^ Both binding modes at the level of DNA affect gene expression: the former through epigenetic modifications, the latter through the recognition and binding of protein factors on specific nucleotide stretch (often consisting of palindromic sequences of at least 12 nucleotides).^17−20^

The impairment of either process, the modulation of histones modifications, or the recruitment time- and space-specific of protein complexes on their nucleotide-binding site, is often involved in the onset of particularly serious pathologies.^21−23^ Already in 1940, the biologist Conrad Hal Waddington, introducing the term epigenetics, emphasized the importance of the interactions of genes with the surrounding environment for the understanding of a specific phenotype. Hence the need to elucidate the mechanisms underlying these complex phenomena and the development of techniques to be applied for their investigation.^24,25^ Therefore, in these past years, we have assisted in the implementation of a large number of analytical methods for studying both the profiling of epigenetics modification in different conditions (i.e., physiological vs pathological)^26−29^ and the isolation and identifications of specific DNA/RNA binding protein complexes.^30−33^

The highest number of experimental approaches, either analytical or preparative, both *in vivo* and *in vitro*, were developed to isolate and identify DNA/RNA binding protein complexes by tacking advantage from their affinity toward a specific oligonucleotide sequence. In proteomics experimental workflows, these affinity-based isolation methods are usually coupled to advanced mass spectrometry (MS) methodologies, for protein identification.^34,35^

Nowadays, many strategies have been borrowed from molecular biology protocols and then improved for high-throughput -omics approaches. Many of them rely on *in vitro* investigations, such as affinity purification and electrophoresis mobility shift assay (EMSA). Conversely, chromatin immunoprecipitation (ChIP) based protocols aim for the isolation and identification of both protein partners and nucleic acid targets of a specific protein “bait” in ex vivo approaches. Finally, newborn techniques based on CRISPR-Cas9 technology have been set up for the isolation and identification of proteins interacting with a specific genomic locus *in vivo*. For each mentioned strategy, several variants have been finely tuned to respond to specific biological problems and to be successfully applied for RNA binding protein investigation fields, too. This review provides an excursus on the main strategies developed in the field of protein–nucleic acid interactions: starting from classical biochemical and/or molecular biological approaches, we focused mainly on those, which coupled with mass spectrometry methodologies, have found the largest applications in the field of -omics sciences. For each approach, the points of strength and drawbacks will be critically treated.

## 2 Probing DNA/RNA–Protein Interactions by *in Vitro* Affinity Procedures

**Figure 1:**
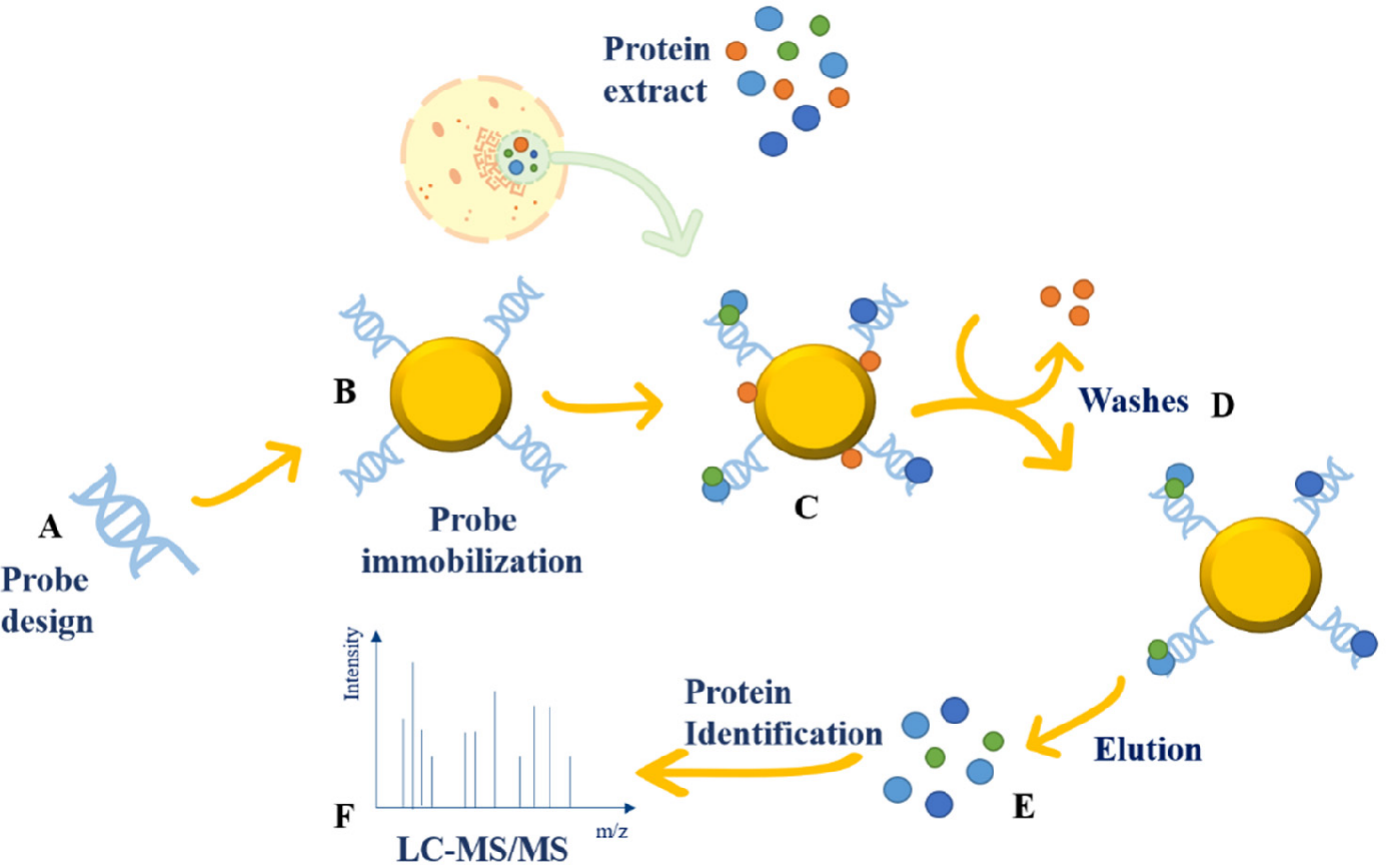
Schematic representation of DNA-pull-down assay workflow. (A) Probe design and synthesis. (B) Chemical or affinity immobilization of the probe on an insoluble solid support. (C) Incubation of nuclear extract with specific oligonucleotide for partners isolation. (D) Washings to remove unspecific proteins. (E) Elution of oligonucleotide interacting proteins. (F) Protein identification by mass spectrometry-based approaches.

The unbiased procedure defined as affinity purification–mass spectrometry (AP-MS) is based on the combination of affinity isolation strategies with MS procedures and constitutes an important branch of the functional proteomics approach.^36−38^ The AP-MS procedure exploits the intrinsic affinity of DNA/RNA binding proteins for a specific oligonucleotide sequence which is used as bait to fish the protein partners out from the cellular extract. In a generic protocol, the protein extract is incubated with the oligonucleotide bait immobilized onto insoluble support. Following several washes proteins, specifically retained by the bait, are eluted and identified by mass spectrometry (Figure 1).

The crucial point in the entire procedure relies on the design of the nucleotide fragment. The length of the probe is chosen according to the number of nucleotides composing the sequence of interest. An optimal bait length is difficult to fix. It would strictly depend on the aim of the study in terms of how narrow the fishing has to be carried out. Generally, longer probes that also include sequences outside the binding region might increase the retention of unspecific proteins lacking the affinity for the consensus sequence. Nevertheless, a longer probe including different regulatory regions can contribute to a cooperative and more stable interaction between DNA/RNA and large multiprotein complexes.^38−40^ On the contrary, too-short probes might affect the stability of protein binding. In order to improve the interaction of the bait with transcriptional factors, which generally are under-expressed and only transiently bind their nucleic acid targets, the oligonucleotide consensus sequence can be multimerized to provide multiple copies of the specific sequence thus increasing the number of potential interacting regions onto the same bait.^41^ In addition, for both DNA and more frequently for RNA probes, the establishment of secondary stable structures have to be considered. Finally, in the design of the oligonucleotide target, the presence of a spacer sequence to properly outdistance the solid support and the binding site of interest, reducing the steric hindrance, has also to be taken into account.^42^ Several procedures have been explored to bind the oligonucleotide bait to the polymeric support, with two methods being the most successful. The nucleotide sequence can be covalently bound by chemical cross-links on a suitably derivatized resin;^43^ alternatively, the oligonucleotide bait can be labeled with biotin and immobilized on streptavidin-coated beads.^44−46^ Binding buffer composition also exerts a critical role in favoring specific DNA–protein interactions. Physicochemical characteristics including pH value, the concentration of mono- and bivalent ions, the addition of specific DNA competitors to reduce nonspecific binding (poly dI, AD, etc.), and finally the amount of glycerol have to be strictly controlled.^47^

The occurrence of nonspecific interactions constitutes a not negligible drawback, which must be faced using the following precautions: (i) stringent washes using at high salt concentrations, (ii) oligonucleotide competitors, like salmon sperm, lacking the target sequence and able to capture background proteins,^48,49^ and (iii) a precleaning phase. The latter relies on a preincubation of protein extract with a not specific oligonucleotide showing the same nucleotide composition of the bait, but modified in specific sites or simply with a random sequence.^38^ However, the discrimination between the true and false interactors is a longstanding central theme in all applications of functional proteomics. More recently, quantitative mass spectrometry-based approaches relied on label-free,^35^ stable isotope labeling by amino acids in cell culture (SILAC),^50^ or tandem mass tag (TMT)^51^ quantification methodologies have been employed to distinguish random interactions from effective ones. In particular, the false positives are expected to be equally abundant in both conditions, while the true binders will be quantitatively prevalent in the presence of the bait.^52^ Finally, software such as CRAPome can provide information on proteins more frequently identified as background in each specific affinity purification experimental system as well as in different cell lines, allowing the recognition and, therefore, the elimination of proteins considered false positives.^38,53^

The “fishing” strategy has found great application in studies addressed to the isolation and identification of nucleic acid-binding proteins involved in fundamental biological processes such as transcription, translation, and/or splicing regulation.^54−57^ In one of the first application, indeed, Medugno and collaborators identified the interaction between the negative cis-element (*AldA-NRE*) and *ZNF224*, a Kruppel-like zinc finger transcription repressor factor as a key step in modulating transcription of the human and mouse aldolase A (*AldA*) gene during the cell cycle and differentiation.^58^ Since then, affinity purification strategies have been further developed and largely applied to the investigation of several biological processes involving key DNA–protein interactions leading to the discovery of previously unknown crucial DNA binding proteins. The nature of the regulatory complex bound to the proximal promoter region to regulate *EPHX1* expression was explored using a biotinylated oligonucleotide encompassing this region in conjunction with mass spectrometric analysis. A 4-component regulatory complex including the inhibitory factor *PSF*, *CAR*, *RXR* and *HNF-4α* was identified.^59^

Nasrullah et al. identified tripartite motif-containing protein 25 (*TRIM25*) as a Caspase-2 mRNA-binding protein in colon carcinoma cells. *TRIM25*, known to be an E3 ubiquitin ligase, seems to exert important tumorigenic functions, controlling metastatic gene signatures both at the transcriptional and post-transcriptional levels. In particular, although *TRIM25* lacks any typical RNA binding domains (RBDs), it can bind and affect the processing and stability of the specific mRNA, acting as a negative regulator of caspase-2 translation. By unveiling this *TRIM25* unexpected function, a novel mechanism of drug resistance in human colorectal carcinoma cells associated with the activity of *TRIM25* as an inhibitor of chemotherapeutic drug-induced apoptosis was described.^60^

Analogously to DNA, RNA molecules, interact with proteins to perform a variety of functions in living cells. Pisa et al. employed AP-MS to isolate novel cap-binding factors that might be involved in translational control of specific mRNAs within the growing Drosophila oocyte, by using m7GTP- derivatized Sepharose beads as bait. About 30 putative interactors were identified, including *Hsp90* which was able to bind the translational repressor Cup *in vitro*, suggesting for Cup novel multiple functions during egg chamber development during *Drosophila* oogenesis.^61^

RNA pull-down experiments in which the RNA bait was covalently immobilized using adipic acid dehydrazide derivatized beads were applied to the identification of specific *ENPP1*-3′ UTR binding proteins revealing that *N*-acetylgalactosaminyltransferase 2 gene (*GALNT2*) was a novel factor involved in the modulation of *ENPP1* expression as well as insulin signaling and action in human liver HepG2 cells.^62^ The same adipic acid dihydrazide-agarose beads were used to bind *in vitro* transcribed RNA probes containing either the wild type or mutated sequence to explore the molecular mechanisms underlying splicing defects in the *DMD* gene. Incubation with a cellular extract followed by mass spectrometry analyses identified proteins that display differential binding affinities for the wild type and mutant RNA probes.^63^

Slightly different variants of the AP-MS approach were designed to address specific biological problems involving functional RNAs. Ray and collaborators introduced AptA–MS (aptamer affinity–mass spectrometry), a robust strategy involving a specific, high-affinity RNA aptamer against green fluorescent protein (*GFP*) to identify the interactors of a GFP-tagged protein. This approach led to the identification of molecular chaperones and translation elongation factors that interact with human heat shock factor 1 (*HSF1*). This technique provides a significant enrichment in terms of sensitivity, in evolutionarily different organisms, and allows identification of PTMs without the need for specific enrichments.^64^

The identification of the protein partners of bacterial small noncoding RNAs was performed by an optimized affinity chromatography protocol that enables purification of *in vivo* formed sRNA–protein complexes. The desired sRNAs were tagged with the MS2 aptamer which is affinity-captured by the MS2-MBP protein conjugated to an amylose resin leading to the recovery of the RNA chaperone Hfq associated with the strictly Hfq-dependent AbcR2 trans-sRNA.^65^

An RNA/DNA hybrid formed by the In-1 transcript and a 5′-biotinylated DNA oligonucleotide complementary to the upstream exon sequence was used to probe HeLa nuclear extracts for spliceosome investigation. The hybrid probe bound with the nuclear proteins was coupled to streptavidin magnetic beads and the retained proteins were identified by mass spectrometry highlighting the occurrence of canonical spliceosome core components belonging to the spliceosomal B-complex.^66^ Analogously, a sequence-specific biotinylated peptide nucleic acid (PNA)-neamine hybrid targeted to HCV RNA was developed for the in situ capture of cellular and viral factors associated with HCV leading to the identification of both cellular factors including transcriptional regulators, RNA helicases, DEAD-box proteins, and translational regulators and three viral proteins (*NS5B*, *NS5A*, and *NS3–4a* protease-helicase) associated with the viral genome.^67^

Recently, G-quadruplex has also been used in pull-down experiments. Guanine quadruple helices, or G-quadruplex, are guanine tetramers stacked together forming a helical structure within the DNA/RNA molecule. They spontaneously form in guanine-rich regions of DNA/RNA giving rise to a variety of conformationally different quadruple helices depending on various factors. G-quadruplex motifs are known to be involved in several biological processes including the mechanisms of initiation of DNA replication and the maintenance of genomic stability by interacting with proteins like chaperones and DNA helicases. Santi Mestre-Fos et al. used G-quadruplex bait in pull-down experiments to identify proteins that specifically recognize these structures in rRNA and applied the SILAC approach for protein quantification. They identified several G-quadruplex linked proteins including helicases (*DDX3*, *CNBP*, *DDX21*, *DDX17*) and heterogeneous nuclear ribonucleoproteins.^68^

Tatsuo Serikawa and collaborators performing pull-down and SILAC experiments identify and quantify proteins that have an affinity for four different G-quadruplex motifs located in mRNAs of the cancer-related genes *Bcl-2*, *NRAS*, *MMP16*, and *ARPC2*. Some of the proteins identified by mass spectrometry appear to be involved in processes of the fine-tuning of translation but are also relevant to the regulation of mRNA maturation and may play an important role in tumor biology.^33^

Although the identification and quantification of DNA/RNA interacting proteins are necessary to understand the biological role of these associations, also the strength of the interactions plays a central role in protein complex characterization. Dissociation constants (*K*_d_) of *in vitro* one-by-one interactions are usually measured by classic biochemical analyses (i.e., isothermal titration calorimetry, surface plasmon resonance, fluorescence polarization, fluorescence resonance energy transfer). Makowski et al. proposed an innovative assay in which DNA affinity purification was coupled with tandem mass tag (TMT) labeling to measure the apparent *K*_d_^App^ values for the identified interactors in pull-down experiments carried out at different bait concentration.^51^ They calculated the protein DNA-bounded fraction by comparing the ion signal of each protein in a single pulldown with that recorded in saturation condition of oligonucleotide bait (micromolar concentration). The *K*_d_^App^ values were calculated by plotting DNA concentration and the bound fractions in a Hill-like curve. The curve also allowed to filter out nonspecific interactions, since background proteins would display randomly distributed signal ratios near 1:1 for all titration points in comparison to control pulldown.

## 3 Electrophoresis Mobility Shift Assay Mass Spectrometry (EMSA-MS)

Since 1981, the electrophoresis mobility shift assay (EMSA) on either polyacrylamide or agarose gel has constituted the most largely employed biochemical procedure to detect *in vitro* DNA–protein interactions, for the simplicity of the procedure, its low cost, and the speed of execution.

The binding of a specific protein to a stretch of DNA (probe) can easily be verified by monitoring the delay (shift) of the oligonucleotide probe following its binding to proteins in comparison with the free probe on a native electrophoretic gel. Moreover, these experiments can also monitor the formation of multiple component complexes simply by observing the supershift originated in the gel electrophoresis by the addition of protein components one at a time.^69^ In the beginning, the electrophoresis shift was usually detected either by using P32 radiolabeled oligonucleotide probes^70−72^ or staining the gel with ethidium bromide.^73,74^ However, nowadays, these methods have completely been replaced by fluorophores (Cy3, Cy5)^75^ or biotin tagged nucleotides^76^ or by the employment of fluorescent dyes.^77^

**Figure 2:**
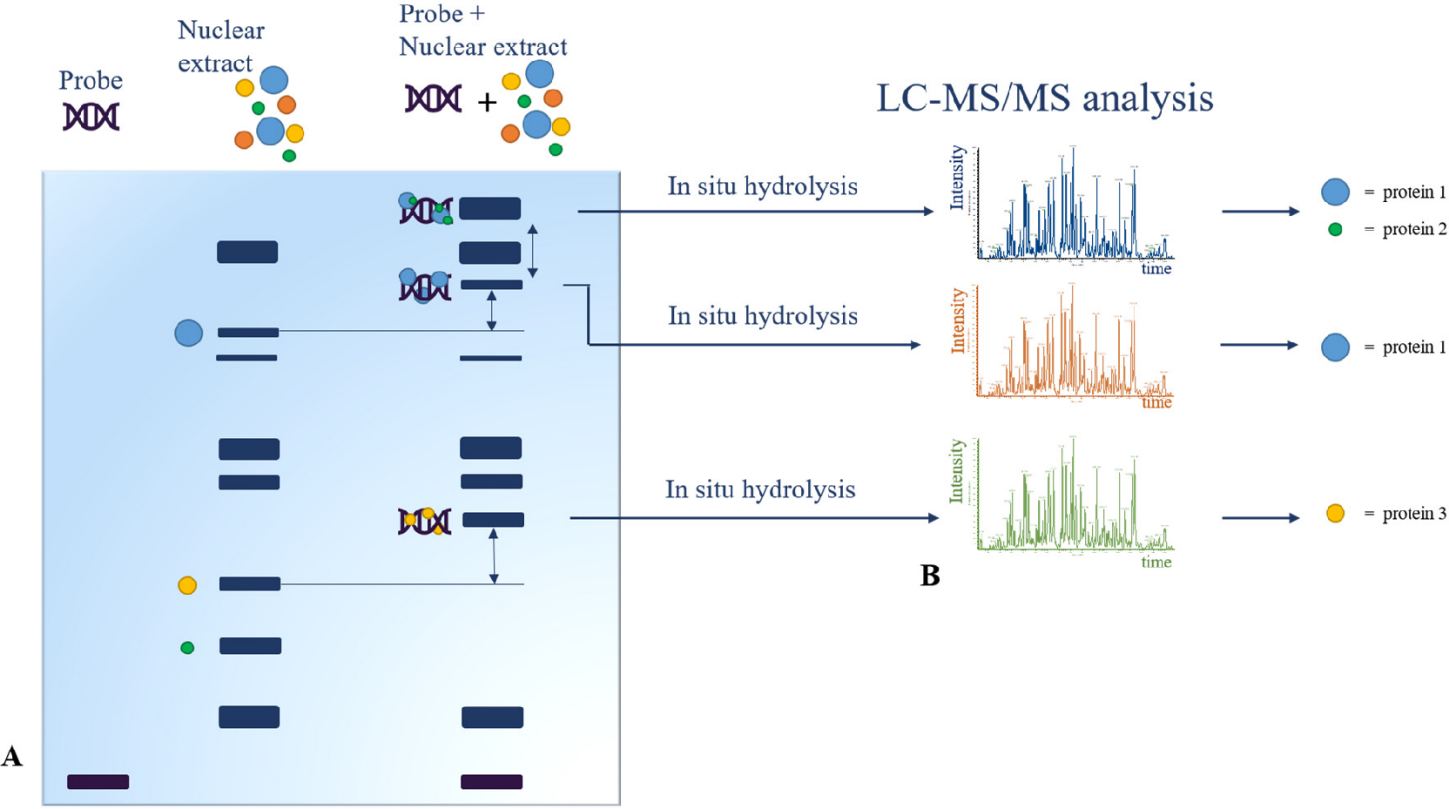
Schematic representation of EMSA-MS experiment. (A) Nuclear proteins are incubated with an oligonucleotide probe and bands showing an electrophoretic mobility shift in comparison to the control are in situ hydrolyzed by trypsin and (B) proteins identified by LC-MS/MS approach. Here the control is the nuclear extract alone (second lane).

In the EMSA classical approach, a recombinant and purified form of the putative DNA- binding protein is incubated with the probe and the interaction is confirmed by the occurrence of a shift in electrophoretic mobility. Alternatively, the protein extract can be challenged with the oligonucleotide probe, and, in the presence of a mobility shift, a specific antibody is further added to confirm the identity of the DNA binding protein. In both cases, knowledge of the protein under investigation is a crucial prerequisite.^78^ More recently, an unbiased approach was developed by coupling the classic EMSA assay with advanced mass spectrometry methodology for the identification of DNA-binding proteins (EMSA/MS). The EMSA/MS procedure combined the simplicity and effectiveness of the EMSA experiments with the ability of high sensitivity, high-resolution mass spectrometry to identify all the proteins interacting with the probe in an unbiased operative mode. Following the EMSA experiment, the shifted band containing the probe-protein complex is excised from the gel and the protein components are identified by tryptic digest and nanoLC-MS/MS analysis of the resulting peptide mixture (Figure 2). Although only a tiny amount of material is usually employed in the EMSA assay, identification of probe binding proteins is quite straightforward due to the high sensitivity of modern mass spectrometers.^79,80^

The focal point of the entire procedure is the choice of the correct negative control to discriminate effective probe binding proteins from false positives, i.e., proteins that display the same electrophoretic mobility of the probe-protein complex. This is especially true when an entire cellular extract is incubated with the probe. A convincing negative control can be obtained by loading the same amount of protein extract on a separate gel lane in the absence of the probe or using an oligonucleotide of the same length and a randomized sequence. For each band excised from the sample lane, an analogous band with the same electrophoretic mobility is picked up from the control lane. Proteins identified both in the sample and the control are discarded.^81^

Möller and co-workers used the electrophoretic mobility shift assay in combination with mass spectrometry to elucidate the molecular genetic bases of the last unresolved blood group system by identifying proteins able to bind the enhancer region rs311103G of the *Xg*^*a*^ human allele. Mobility shifts were noted after the addition of a nuclear extract to the oligonucleotide and the individual components in the probe/protein complexes were identified by tandem LC-MS/MS leading to the identification of *GATA1* protein.^82^

Analogously, EMSA/MS was performed to identify proteins binding to the *ZNF423* single nucleotide polymorphisms (SNP), a potential biomarker for response to selective estrogen receptor modulators (SERMs) therapy for breast cancer prevention. After EMSA, the shifted gel bands indicating specific DNA–protein interactions were isolated and submitted to mass spectrometry analysis identification. Calmodulin-like protein 3 (*CALML3*) was identified as a key sensor of this SNP and a coregulator of ERα, which contributes to differential gene transcription regulation in an estrogen and SERM-dependent fashion.^83^

A slightly different approach was used by Fusco et al. in their investigation on proteins associated with *F55*, a transcription repressor belonging to *S. solfataricus* (*S.so*) spindle-shaped virus 1 (*SSV1*) when the transcription factor is bound to its specific DNA promoter sequence. When the probe was incubated with the *S.so* protein extract containing *F55*, two different delayed bands were clearly detected by fluorescence. Western Blot assay revealed the presence of *F55* in only one of the two bands that were in situ digested with trypsin and the proteins identified by a nanoLC-MS/MS-based strategy. Among the putative *F55* interactors, *RadA*, a homologue of *E. coliRecA*, was identified, suggesting that the archaeal molecular components *F55* and RadA are functional homologues of bacteriophage λ (factor *CI*) and *Escherichia coli* (*RecA*) system.^84^

## 4 Chromatin Purification Methods Coupled with Mass Spectrometry

**Figure 3:**
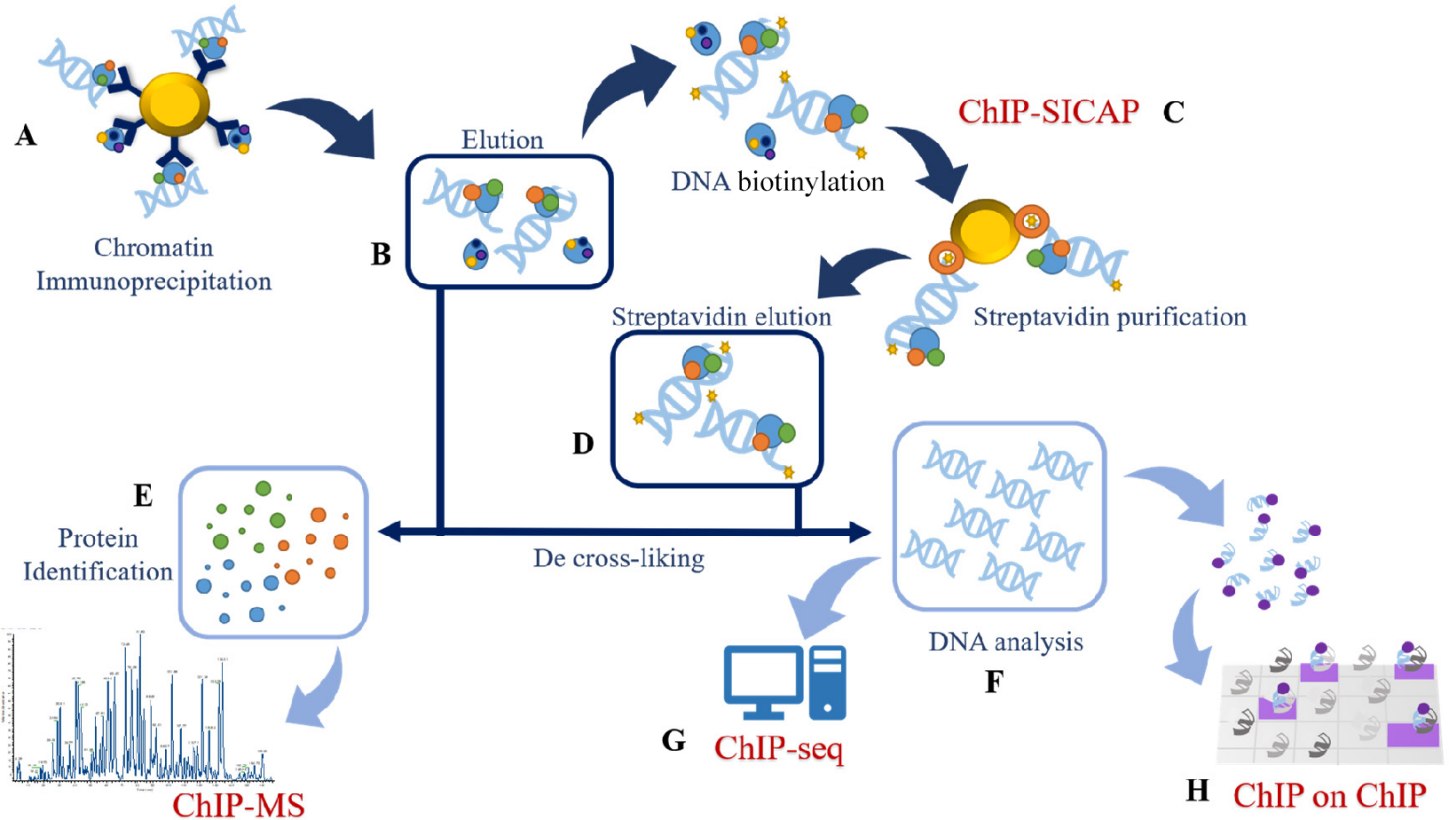
A generic representation of ChIP experiments. (A) After chromatin immunoprecipitation, (B) DNA–protein complexes are eluted. (C) For only DNA–protein complex isolation, ChIP-SICAP experiment is possible by DNA biotinylation and streptavidin purification, and then (D) elution and de-cross-linking. Finally, protein identification (E) was carried out by mass spectrometry approach (ChIP-MS) and DNA analysis (F), by (G) sequencing (ChIP-seq) or (H) by hybridization with a pull of fluorescence probe (ChIP on ChIP).

Despite a considerable amount of biochemical data obtained with *in vitro* experimental systems, detailed information about the interactions between transcription factors and their targets *in vivo* has been obtained following the introduction of chromatin immunoprecipitation (ChIP) technique (Figure 3).^85^ According to ChIP protocol, the *in vivo* interactions between transcriptional factors or other DNA binding proteins are stabilized by chemical cross-links. The protein–DNA complexes are then immunoprecipitated using a specific antibody for the protein of interest following sonication. In a classical approach, proteins are completely digested and the immunoprecipitated DNA regions are identified by PCR amplification and/or DNA sequencing (ChIP-seq) (Figure 3G).^86^ The ChIP procedure combined with the knowledge of the human genome opened incredible new scenarios allowing the researchers to deeply understand how transcriptional factors affect crucial cellular processes through the identification of their target genes.^87,47^

As well as for AP-MS, even for a ChIP experiment, a control experiment must be designed. It could consist in the use of the solid support only, called “beads-only” control, which does not involve antibodies and provides solely the nonspecific adsorption due to the beads used for the experiment.^88^ A more stringent control includes a nonspecific antibody, to be used in a parallel ChIP experiment, as that employed by Collas and collaborators to demonstrate the binding specificity of *Oct4* on the *NANOG* promoter in pluripotent carcinoma cells.^88^

Since the introduction of the ChIP technique, new upgrades have been developed like the Re-ChIP method, in which two antibodies directed against two different antigens are used, one after the other, allowing the identification of DNA fragments where two protein factors are simultaneously bound;^89−92^ or the ChIP-on-ChIP variant, in which immunoprecipitated DNA is used as a probe to hybridize a slide containing fragments of genomic DNA (chips) leading to the identification of new targets (Figure 3H). Initially developed in yeast, ChIP-on-ChIP is today successfully applied to human systems.^93−96^ As mentioned above, the ChIP procedure constituted a fantastic improvement in the investigation of DNA–protein interactions both *in vitro* and *in vivo*. However, although this approach was born to be addressed to the identification of DNA target regions where a specific protein factor is bound to, in the last years, the attention was moved also toward the investigation of the additional proteins contemporarily present on the same oligonucleotide sequences. Nowadays, it is well-known that DNA binding proteins are embedded within multiprotein complexes to fulfill their biological role. No information can be provided by the classical ChIP procedures on the other individual components of these functional complexes. Only recently, with the advent of functional proteomics, a fundamental modification of the ChIP procedure, defined ChIP-MS^97,98^ or rapid immunoprecipitation mass spectrometry of endogenous protein (RIME) has been proposed, with the aim to provide simultaneous identification of the specific DNA target sequences and the protein components of the functional complex bound to those regions.^99−101^

According to these aims, the ChIP protocol was adapted to allow protein identification. The DNA binding protein complex is allowed to bind its DNA target sequence *in vivo* and then covalently cross-linked to the DNA stretch. The stabilized complex is immunoprecipitated following the classical ChIP procedure and then either enzymatically digested with trypsin directly on the beads (RIME) or eluted, de-cross-linked, and digested with trypsin (ChIP-MS) (Figure 3E). In both cases, the DNA target is amplified for sequencing, whereas the peptide mixture is directly analyzed by nanoLC-MS/MS to provide protein identification.^102^

Hwang and co-workers used the chromatin immunoprecipitation procedure coupled to mass spectrometry (ChIP-MS) to identify β-catenin-interacting proteins within a multifunctional protein that might be involved in transcriptional regulation in rat inner medullary collecting duct (IMCD). Several β-catenin-binding proteins were identified, including several known β-catenin-binding partners as well as novel interacting proteins among which *Taf1*, *Jup*, *Tdrd3*, *Cdh1*, *Cenpj*, and several histones were involved in transcriptional regulation.^103^

A large systematic investigation of proteins that bind transcriptional enhancers and promoters in embryonic stem cells was carried out by exploiting chromatin immunoprecipitations (ChIP) by using antibodies for characteristic histone modifications and identification of associated proteins using mass spectrometry.^104−107^ The ChIP-MS method provided a detailed read-out of the transcriptional landscape representative of the investigated cell type, leading to the identification of several protein factors, most of which drive reprogramming to pluripotent stem cells^100^ such as *Oct4*, *Esrrb*, *Klf5*, *Mycn*, and *Dppa2*.

A functional proteomic experiment based on ChIP-MS was designed to understand the molecular mechanisms that underlie the involvement of *CBX7* in cancer progression. *CBX7*, a component of the polycomb repressive complex (*PRC1*), can positively or negatively regulate the expression of genes involved in cell proliferation and cancer progression, including E-cadherin. Using the ChIP-MS approach, Federico et al. demonstrated that *CBX7* effectively interacts with histone deacetylase 2 (HDAC2)^92^ and with protein arginine methyltransferase 1 (*PRMT1*)^91^ on the E-cadherin promoter. These findings demonstrated that *CBX7* activity on E-cadherin promoter is strictly related to several enzymes involved in epigenetic modifications and belonging to both so-called writers (i.e., *PMRT1*) and erasers (i.e., *HDAC2*) categories.

The main drawbacks in the ChIP-MS procedure lay in a large number of contaminants due to the possible capture and identification of nonchromatin-associated complexes like those involving proteins or transcription factors that form different complexes on and off chromatin in response to different stimuli. Rafiee et al. proposed a modified version of the ChIP-MS protocol to specifically identify proteins in their DNA-bound state. The method combines ChIP with selective isolation of chromatin-associated proteins (SICAP) (Figure 3C) followed by mass spectrometry to identify chromatin-bound partners of a protein of interest. DNA protein complexes are cross-linked by formaldehyde, and fixed chromatin is immunoprecipitated with a suitable antibody and fragmented by sonication. DNA fragments are then biotinylated and chromatin is retrieved along with interacting proteins on streptavidin beads. Following extensive washing, the cross-link is reversed and proteins are trypsin digested and identified by mass spectrometry.^108^

The effectiveness of ChIP-SICAP was demonstrated by characterizing the chromatin-bound network around *Oct4*, *Sox2*, and *Nanog* in mouse ESCs, the so-called OSN system, leading to the discovery of *Trim24* as a component of the pluripotency network. ChIP-SICAP uniquely benefits from the double purification of protein–DNA complexes, accomplished by subsequent ChIP of the protein of interest and pull-down of biotinylated DNA allowing the exclusive capture of protein complexes bound to DNA.^109^

In general, ChIP based methods consist of protein-centric approaches since the purification strategies described are tailored according to the identity of the protein of interest that interacts with DNA/RNA. Conversely, alternative DNA/RNA-centric approaches have been developed in order to isolate protein complexes by fishing them from the specific genomic locus. In the proteomics of isolated chromatin segments (PICh)^20^ strategy, the formaldehyde-cross-linked protein/chromatin complexes are isolated through nucleic acid hybridization and identified by mass spectrometry methodologies. A specific capture probe called locked nucleic acid (LNA), consisting of an RNA nucleotide sequence in which the ribose moiety is modified with an extra bridge connecting the 2′ oxygen and 4′ carbon with increased 3′-exonucleolytic stability and improved hybridization affinity,^110^ is employed for soluble chromatin hybridization and then for the isolation of the specific genomic locus of interest. The probe is also tagged with a desthiobiotin that allows its affinity purification.

Déjardin et al. developed and used the PICh strategy to purify and analyze protein complexes bound to two distinct types of the telomere, as a proof-of-principle, in three human cell lines: *HeLa S3*, *HeLa 1.2.11*, and *WI38-VA13 ALT*. By designing a specific probe for the PICh experiment and a scrambled probe as control, they were able to isolate the telomeric regions of interest and identified 85% of the proteins known to be associated with them.^20^

Additional analogous strategies for comprehensive identification of RNA-binding proteins (ChIRP), in capture hybridization analysis (CHART-MS)^111,112^ or RNA antisense purification (RAP-MS)^113^ were set up to isolate RNA or ncRNA binding proteins, by employing DNA (for CHART, ChIRP) or transcribing DNA into RNA (for RAP)^114^ hybridization capture-based approach. The main difference among these techniques consists in probe design: in ChIRP and RAP techniques, a pool of oligonucleotides that cover the full length of the RNA target is used, while in CHART-MS only a few shorter probes are required.^114,115^*In vivo*, RNA–protein interactions are chemically cross-linked and purified using biotinylated oligonucleotides complementary to the RNA stretch of interest. Coprecipitated proteins are eluted and identified by mass spectrometry. This approach resulted effective for both abundant housekeeping and relatively low expressed RNAs.^116,117^ ChIRP-MS analysis allowed the identification of multiple splicing factors in nuclear stress bodies (nSBs) containing long noncoding RNAs, including serine and arginine-rich pre-mRNA splicing factors (SRSFs) which affect splicing patterns.^118^ The same strategy was employed to explore carcinogenic mechanisms involving long noncoding RNA SNHG6 in colorectal carcinoma onset, leading to the identification of several proteins involved in spliceosomes and mRNA processing.^119^

CHART-MS strategy was used by West et al. to elucidate genomic binding partners of two human lncRNAs, *NEAT1* (nuclear enriched abundant transcript 1) and *MALAT1* (metastasis-associated lung adenocarcinoma transcript 1). In this study, they showed that *NEAT1* and *MALAT1* bind multiple active genes. The two lncRNA, when colocalized on the same DNA region, displayed different protein partners indicating different but synergistic functional roles.^111^

RAP (RNA antisense purification) is a biochemical method described in 2015 by Engreitz and co-workers^120^ that enables mapping of RNA interactions with chromatin. As for other several examples reported in this review, in its original version, this approach aimed for the identification of DNA loci interacting with the target RNA by using high-throughput DNA sequencing. Three years later, the same research group proposed an upgrade of the method called RAP-MS, in which the RNA antisense purification was coupled with mass spectrometry for the identification of proteins directly interacting with a specific RNA molecule.^113^

RAP-MS strategy uses ultraviolet light to cross-link and stabilize only direct protein interactions and is coupled with SILAC protein quantification.^121^ Wanowska et al. investigate the effect of *ENST00000501665.2*, *OIP5-AS1* (OIP5 Antisense RNA 1) splicing variant, on Opa interacting protein 5 (*OIP5*) expression with RAP-MS strategy. In HEK293 cells, they demonstrate that ENST00000501665.2 is a positive regulator of *OIP5* expression by binding *SMARCA4*, a component of the SWI/SNF complex and facilitating the interaction between SWI/SNF chromatin remodeling complex and *OIP5* promoter.^122^

## 5 CRISPR-Based Chromatin Affinity Purification–Mass Spectrometry (CRISPR-ChAP-MS)

Recently, another locus-specific strategy exploits the CRISPR (regularly clustered interspaced palindromic repeats)/dCas9 system has been developed. A specific locus is targeted using nuclease-deficient Cas9 (dCas9) (often labeled with a terminal protein or peptide tag, e.g., FLAG, myc, etc.) in combination with a specific guide RNA (gRNA). Normally, the gRNA is designed to bring the dCas9 upstream to the locus of interest. Then, an experiment resembling chromatin immunoprecipitation is carried out: following the cross-linking and sonication procedure, dCas9 is immunoprecipitate bound to chromatin through the gRNA anchor. De-cross-linked proteins are then identified by mass spectrometry methodologies in a combined approach known as CRISPR-ChAP-MS.^123^ In the latter, the optimal experimental control is provided by the same cell line transfected with dCas9 in the absence of gRNA.

**Figure 4:**
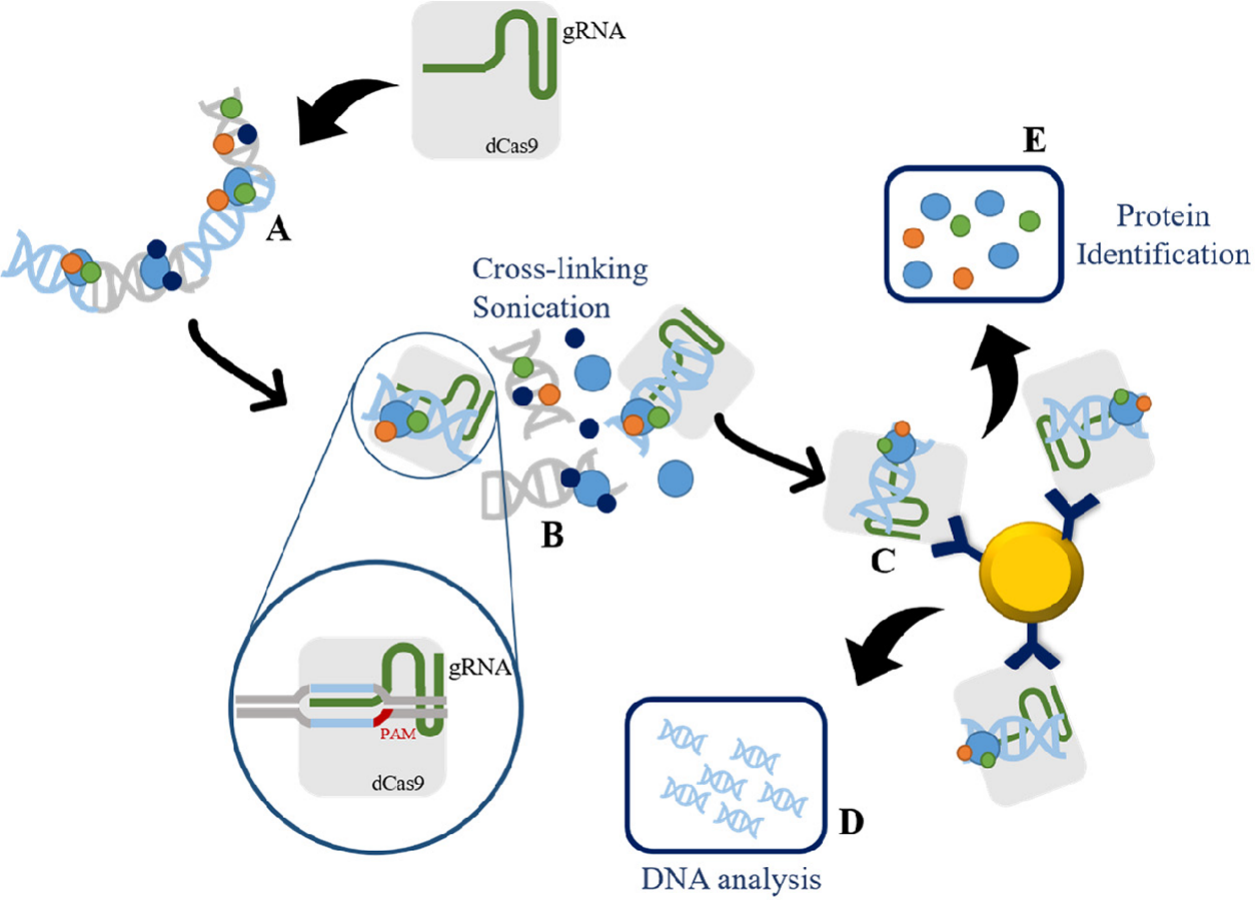
Schematic workflow of CRISPR-ChAP-MS experiment. (A) Overexpression of dCas9 and a specific RNA guide (gRNA), (B) binding to the specific promoter, cross-linking and sonication. (C) Immunoprecipitation of Cas9 for the isolation of DNA–protein complexes of the promoter of interest. After de-cross-linking, DNA (D) and protein (E) elution are carried out.

Waldrip et al. introduced the CRISPR-ChAP-MS approach to isolate the protein complex specifically linked to the *GAL1* promoter in yeast under transcriptionally active conditions. Cells were treated with formaldehyde to stabilize protein–DNA interactions, chromatin was sheared to fragments, and the target chromatin region harboring the *Gal1* promoter was specifically located using the CRISPR-Cas9 system. The complex was affinity purified by a Protein A tagged version of *Cas9* together with the proteins gathered at the promoter that was then eluted and identified by mass spectrometry^123^ (Figure 4).

The affinity enrichment strategy CRISPR-ChAP-MS was used to define how transcription from the arsenic response locus is regulated in an arsenic-dependent manner in budding yeast.

This locus constitutes a conserved pathway ranging from prokaryotes to higher eukaryotes. CRISPR-ChAP-MS was applied to the promoter regions of the activated arsenic response locus and the proteomic characterization of the targeted protein complex uncovered 40 nuclear-annotated proteins. Among these, the histone acetyltransferase SAGA and the chromatin remodeling complex SWI/SNF required for the locus activation were identified, providing key insight into the mechanisms of transcriptional activation required for the detoxification of arsenic from the cell.^124^

CRISPR-ChAP-MS was also successfully applied for application in mammalian cell models.

*MACC1* (colon cancer-associated metastasis 1) is a protein that induces metastasis in colon cancer. The mechanisms by which its expression is transcriptionally regulated is still not fully known. Huang and collaborators exploited the CRISPR-ChAP-MS technique to identify proteins interacting with *MACC1* promoter. They use the catalytically inactive 3xFLAG tagged dCas9 along with a gRNA to target and isolate the promoter region of the *MACC1* gene.^125^ The c-JUN transcription factor was found physically bound to the *MACC1* promoter and able to upregulate its expression.

In addition to the basic approach, many following versions have been enriched with further experimental steps and then have been proposed. For instance, a new hybrid approach (CasID)^126^ was introduced by Schmidtmann et al. and was based on BirA* and dCas9 to label by biotinylation protein components present at specific DNA sequence within a 10 nm range, thus to map also transient interactions. By using the construct BirA*-dCas9-eGFP, Schmidtmann and collaborators identified, for example, TERF2, TINF2, ACD that are known to directly bind telomeric DNA but also new chromatin factors, demonstrating that CasID is a robust method to investigate native protein environment at specific genomic loci. This approach provides a more detailed view of complexes that involve specific chromatin loci under dynamic and functional aspects if compared to ChIP. On the basis of the integration of CRISP technology and the proximity labeling enzyme APEX2 (dCas9-APEX2), Gao and collaborators proposed the new method C-BERST^127^ as an alternative to CasID.

Another valid strategy inspired by CRISPR-dead Cas9 was introduced by Yi and collaborators and was called CARPID^128^ (CRISPR-assisted RNA–protein interaction detection). It has been proposed to overcome the limitations of many other techniques used to elucidate the interactions between lncRNAs (noncoding RNA consisting of more than 200 nucleotides in length) and proteins in living cells. They designed a gRNA array composed of two gRNA sequences spaced by a 30-nucleotide direct repeat to target 2 adjacent loci on the same lncRNA transcript which could offer both greater specificity of targeting and a reduction in background noise. Yi et al. used an X-inactive specific transcript (*XIST*), a well-studied mammalian lncRNA, to validate the efficiency of the method. Besides already known protein partners, several novel *XIST*-interacting proteins have been identified; in particular, *TAF15* (transcription activators) and *SNF2L* (repressive factors) that confirmed previous models of *XIST*-mediated X chromosome inactivation (XCI). This technique allowed them to confirm that it is an excellent method for identifying RNA–protein interactions but still has limitations and should be considered complementary and associated with other types of investigations.^128^

More recently, another Cas enzyme, i.e., *Cas13*, has been introduced to purify RNA-specific targets and identify proteins associated with an endogenous RNA within the RNA proximity proteomic methods (CBRPP),^129^ a new RNA-centric method.

## 6 Conclusions

Over the years, the advancement of technologies and the epochal changes in the way of looking at the biological world typical of -omic approaches have allowed the comprehension of many cell processes as well as the definition of new scenarios hitherto unknown. The combination of functional proteomics experiments coupled with mass spectrometry for the isolation and identification of protein complexes on specific DNA/RNA regions or investigation of epigenetic modifications both as profiling and at the level of gene promoters have had the largest impact in the elucidation of processes concerning gene regulation, splicing, translation, and so on. The findings deriving from these high throughput studies have allowed the understanding of the complex series of events, most of them also involved in the onset of several diseases.

From a methodological point of view, affinity purification (AP), ChIP, EMSA, CRISPR, and their variations provide complementary information.^130−132^ Among these methods, those *in vitro*, such as AP and EMSA, are more approachable and cheaper, although they suffer of all limitations associated with *in vitro* approaches. Others, i.e., DNA/RNA-centered ChIP and CRISPR-based, provide more detailed clues relative to a single genomic locus analysis *in vivo* but require more elaborate cell systems to be developed. Despite that the latter are time-consuming and expensive procedures to be set up, they are the only that allow the investigation of different DNA/RNA interactomes, modulated *in vivo* by different stimuli.^133^ All the described procedures might be enriched by the integration with structural data, such as the analyses of contact interfaces at the amino acid level,^134^ or the identification of post-translational modifications (PTMs) and how they tune the binding to DNA/RNA,^135^ and finally with innovative strategies for the global analysis of all putative DNA/RNA binding proteins.^136^ Overall, mass spectrometry is and could further be a master technology for all the present and future applications in the investigation of the nucleic acid interactome.

## Abbreviation

AP-MS: affinity purification–mass spectrometry
AP: affinity purification
AptA-MS: aptamer affinity–mass spectrometry
*CALML3*: Calmodulin-like protein 3
CHART-MS: capture hybridization analysis of RNA targets–mass spectrometry
ChIP: chromatin immunoprecipitation mass spectrometry
ChIP-MS: chromatin immunoprecipitation–mass spectrometry
CRISPR: clustered regularly interspaced short palindromic repeats
ChIRP-MS: comprehensive identification of RNA-binding proteins–mass spectrometry
CARPID: CRISPR-assisted RNA–protein interaction detection
CRISPR-ChAP-MS: CRISPR-based chromatin affinity purification–mass spectrometry
CRISPR-ChAP: CRISPR-based chromatin affinity purification
*K*_d_: dissociation constants
EMSA: electrophoresis mobility shift assay
EMSA-MS: electrophoresis mobility shift assay–mass spectrometry
*GFP*: green fluorescent protein
*HSF1*: heat shock factor 1
IMCD: inner medullary collecting duct
lncRNAs: noncoding RNA
LNA: locked nucleic acid
*MACC1*: colon cancer-associated metastasis 1
*MALAT1*: metastasis-associated lung adenocarcinoma transcript 1
*GALNT2*: *N*-acetylgalactosaminyltransferase 2 gene
*NEAT1*: nuclear enriched abundant transcript 1
nSBs: nuclear stress bodies
PNA: peptide nucleic acid
PRC: polycomb repressive complex
PCR: polymerase chain reaction
*PRMT1*: protein arginine methyltransferase 1
PICh: proteomics of isolated chromatin segments
RIME: rapid immunoprecipitation mass spectrometry of endogenous protein
RAP-MS: RNA antisense purification–mass spectrometry
RBDs: RNA binding domains
*S.so*: *S. solfataricus*
SERMs: selective estrogen receptor modulators
SICAP: selective isolation of chromatin-associated proteins
SRSFs: serine and arginine-rich pre-mRNA splicing factors
SNP: single nucleotide polymorphisms
*SSV1*: spindle-shaped virus 1
SILAC: stable isotope labeling by amino acids in cell culture
TMT: tandem mass tag
*CBX7*: chromobox protein homologue 7
*TRIM25*: tripartite motif-containing protein 25
ssDNA: single-stranded DNA
SSB: DNA binding proteins
*XIST*: X-inactive specific transcript.
